# PBJ is very thankful for following reviewers who contributed to the peer review process from 1st January 2020 to 31st December 2020

**DOI:** 10.1111/pbi.13553

**Published:** 2021-03-13

Siti Nor Akmar Abdullah

Amina Abed

Nikolai Adamski

Eduard Akhunov

Samir Alahmad

Marc Albertsen

Andrew Allan

Randy Allen

Chuanfu An

Rudi Appels

Tzahi Arazi

Tim Artlip

Sarah Assmann

Jean‐Marc Aury

Brian Ayre

Sangsu Bae

Bochra Bahri

Songling Bai

Yang Bai

Yeming Bai

Jutta Baldauf

Patricia Baldrich

Peter Balint‐kurti

Julia Bally

Surinder Banga

Kailash Bansal

Goran Barac

Rafal Baranski

Piero Barone

Laura Bartley

Filippo Bassi

Jacqueline Batley

Philipp Bayer

Frederic Beaudoin

E. Beaver‐Kanuya

Lidija Begovic

Philip Benfey

Tom Bennett

Christoph Benning

Analia Berinstein

Cinzia Bertea

Scott Betts

Dharminder Bhatia

Navreet Bhullar

Paul R. J. Birch

Chiara Biselli

Sukumar Biswas

Thomas Blein

Ralph Bock

Wout Boerjan

Petra C. Boevink

Luisa Bortesi

Jose Botella

Henrik Brinch‐Pedersen

Anne Brown

Roque Bru‐Martínez

Amy Brunner

Peter Buchner

Hikmet Budak

Steven Burgess

Johannes Buyel

Julieta Cabello

Edgar Cahoon

Dong Cao

Daniela Andrea Capiati

Alberto Carbonell

Florian Cardon

Edward Cargill

Sabine Carpin

Yves Carrière

Stuart Casson

Jorge Alexander Duitama Castellanos

Alexandra Castilho

Amanda Cavanagh

Felice Cervone

David Chagné

SANHITA CHAKRABORTY

Boulos Chalhoub

Zhulong Chan

Debasis Chattopadhyay

Paul Chavarriaga

Baihong Chen

Charles Chen

Chen Chen

Fadi Chen

Fang Chen

Fei Chen

Feng Chen

Feng Chen

Gongyou Chen

Jiansheng Chen

Kunming Chen

Ling‐Ling Chen

Quan‐Jia Chen

Shaojiang Chen

Shaoliang Chen

Sixue Chen

Su Chen

Sumei Chen

Xianming Chen

Xuehao Chen

Xuesen Chen

Xuewei Chen

Lailiang Cheng

Aixia Cheng

Shifeng Cheng

Zhukuan Cheng

Ravindra Chibbar

Joong Hyoun Chin

kang chong

Chengcai Chu

Nam‐Hai Chua

Wu Chuanyin

Stephan Clemens

Tom Clemente

Heather Coleman

Helen Collins

Tony Condon

Lucio Conti

Wallace Cowling

Wayne Crismani

Pierre Crozet

Haitao Cui

Sébastien Cunnac

Josh Cuperus

Rosa M. Cusido

Marc André D'Aoust

Lingling Da

Cheng Dai

James Dale

Henry Daniell

Ruud de Maagd

Antonio De Oliveira

Clelia De‐la‐Peña

Xing Wang Deng

Yiwen Deng

Neetin Desai

Harcharan Dhaliwal

Amit Dhingra

Steve DiFazio

Fang Ding

Jing Ding

Shi‐You Ding

xiaodong Ding

Jose Dinneny

Diaga Diouf

Gianfranco Diretto

Ray Dixon

Richard Dixon

Jingao Dong

Suomeng Dong

Fu Donghui

Daolong Dou

Xiongming Du

Zhi‐Yan Du

Erchao Duan

Xuewu Duan

Mukesh Dubey

Christian Dubos

Stephen Duke

Peter Eastmond

Patrick P. Edger

Christine Edwards

Mohamed El‐Esawi

Neil Emery

Michael Emes

Tara Enders

Masaki Endo

Aymerick Eudes

Michelle Facette

Bryce Falk

Kai Fan

Liumin Fan

Yuanwei Fan

David Fang

Shui‐zhang Fei

Zhangjun Fei

Jialu Feng

Robert J. Ferl

Alisdair Fernie

Cristina Ferrándiz Maestre

Osvaldo Ferrarese‐Filho

John Finer

Matthias Fladung

Elizabeth Fontes

Christine H. Foyer

Paul Fraser

Ursula Frei

Chunxiang Fu

Daolin Fu

Xiangdong Fu

Xiumin Fu

Kazuhito Fujiyama

Takeshi Fukao

Caiji Gao

Caixia Gao

Wei Gao

Roberto Gaxiola

Xianhong Ge

Xiaochun Ge

Stanton Gelvin

Alessandra Gentile

Milen Georgiev

Isabel Gerhardt

Prithwi Ghosh

Donato Giannino

Eleanor Gilroy

Simon Gilroy

Patricia Giraldo

Chloe Girard

Ian Godwin

Michel Goldschmidt‐Clermont

Agnieszka Golicz

Wankui Gong

Nathalie Gonzalez

Tatyana Gorshkova

Charles Goulet

Jonathan Gressel

Rebecca Grumet

Xiaofeng Gu

Yuefeng Guan

An‐Yuan Guo

HuiShan Guo

Liang Guo

Longbiao Guo

Wangzhen Guo

Charles Hachez

Nigel G. Halford

Claire Halpin

Abdelali Hannoufa

Karen Harris

John Harwood

Wendy Harwood

Richard Haslam

Joshua Hazelwood

Guanghua He

Guangyuan He

Shaozhen He

Yuqing He

Zuhua He

Peter Hedden

Kathleen Hefferon

Jan Hejatko

Klaas J. Hellingwerf

Robert Henry

Götz Hensel

Pilar Hernandez

Julian Hibberd

Lee Hickey

Lori Hinze

Arvind H. Hirani

Kendal Hirschi

Hiroshi Hisano

Chikako Honda

Delin Hong

Gaojie Hong

Patrick Horn

Wensheng Hou

Yingnan Hou

Kelly Houston

Guanghui Hu

Lisong Hu

Qiong Hu

Xuehai Hu

Zhaorong Hu

Jian Hua

Xuejun Hua

Fang Huang

Jirong Huang

Junli Huang

Li‐Fen Huang

linkai huang

Sanwen Huang

Sanwen Huang

Xuehui Huang

Matthew E. Hudson

Shou Huixia

Brent Hulke

Heqiang Huo

Mathilde Hutin

Ashraf Islam

Natalia B. Ivleva

Takeshi Izawa

Paula Jameson

Philippe Jeandet

JaeHeung Jeon

Jong‐Seong Jeon

Soon‐Chun Jeong

Haifeng Jia

Yulin Jia

Mao Jianfeng

Lixi Jiang

Yueming Jiang

Yinping Jiao

Shuangxia Jin

Liesche Johannes

Eric Johnson

Paul A. Johnston

Dan Jones

Jeffrey Jones

Christian Jung

Ki Hong Jung

suhas kadam

Schawn Kaeppler

Guoyin Kai

Chutchamas Kanchana‐udomkan

Hong‐Gu Kang

Chunying Kang

Zhensheng Kang

Kostya Kanyuka

Parwinder Kaur

Taiji Kawakatsu

Yoji Kawano

Oliver Kayser

Beat Keller

Hoon Kim

HyunSoon Kim

Jae‐Yean Kim

Sunggil Kim

Yun Ju Kim

Roy Njoroge Kimotho

Graham King

Jaroslav Klápště

Daniel J. Kliebenstein

Steven J. Knapp

Dominic Knoch

Jae‐Heung Ko

Kisung Ko

Hee‐Jong Koh

Fanjiang Kong

Maarten Koornneef

Richard Kormelink

Stanislav Korpiva

Yanjun Kou

Nik Kovinich

Manoj Kulkarni

Shashi Kumar

Aloka Kumari

Yukio Kurihara

Smita Kurup

Peter LaFayette

K Lai

Hon‐Ming Lam

Nicolas Langlade

Laurent Laplaze

Philip Larkin

Fernanda Lazzarotto

Jie Le

Huey Tyng Lee

Keunsub Lee

Scott Lenaghan

Dayong Li

Ai‐Hong Li

Aili Li

Chengdao Li

Chuanyou Li

Chunhui Li

Fengcheng Li

Genyi Li

Genying Li

Guanglin Li

Haifeng Li

Jianfeng Li

Laigeng Li

Li Li

Maoteng Li

Wanlong Li

Xia Li

Xianran Li

Xuan Li

Xuan Li

yi li

Yunhe Li

Zhengguo Li

Zhikang Li

Zhongyi Li

Chengzhi Liang

Wanqi Liang

Hhong Liao

Chentao Lin

Hao Lin

Jeng‐Shane Lin

Wen‐Hui Lin

Yann‐rong Lin

Yongjun Lin

Zhongwei Lin

Zongcheng Lin

John Lindbo

Johannes von Lintig

Hongtao Liu

Yule Liu

Jihong Liu‐Clarke

Bao Liu

Baohui Liu

Chang‐Jun Liu

Changying Liu

Chun‐Ming Liu

Ji‐Hong Liu

Junzhong Liu

Kede Liu

Mingchun Liu

Qiaoquan Liu

Qingchang Liu

Sanzhen Liu

Shengyi Liu

Wusheng Liu

Xiaolei Liu

Xin Liu

Yao‐Guang Liu

Yongbo Liu

Yuqiang Liu

Zhiyong Liu

Cesar Llave

Lu Long

Stephen P. Long

Rosa Lozano‐Duran

Chaofu Lu

Kun Lu

Meng‐Zhu Lu

Shan Lu

Tiegang Lu

Yingtang Lu

Sheng Luan

Yushi Luan

Thomas Lubberstedt

A. J. Lukaszewski

John Lunn

Hong Luo

SENTHILKUMAR K M

Changle Ma

Chuang Ma

Fengwang Ma

Langlang Ma

Ligen Ma

Qi‐Bin Ma

Tao Ma

Wujun Ma

Youzhi Ma

Zhengqiang Ma

Zhiying Ma

Ian Mackay

Fatemeh Maghuly

Magdy Mahfouz

chuanzao mao

Long Mao

Yingbo Mao

Clemence Marchal

David Marshall

Frédéric Marsolais

Federico Martin

Maria‐Jesus Martin

Pedro Martinez‐Gomez

Gustavo Martinez

Carla Marusic

Kyonoshin Maruyama

Esten Mason

Hugh Mason

Sean Mayes

Stephen Mayfield

Mitra Mazarei

Karen McDonald

Richard Meilan

Michael Meissle

Ewa Mellerowicz

DONG MENG

Raphael Mercier

Ángel Mérida

Dominique Michaud

Daisuke Miki

A. Harvey Millar

Tony Miller

Joel Milner

Ling Min

Shakirin Mispan

Ali Missaoui

Rowan Mitchell

Toshiaki Mitsui

Kenji Miura

Yoko Mizuta

Pravat Kumar Mohapatra

João Vitor Molino

Kutubuddin Molla

Attila Molnar

Masaki Mori

Takaya Moriguchi

Gary Muehlbauer

Gary J. Muehlbauer

Oliver Mueller‐Cajar

Helene MURANTY

Yoshiyuki Murata

Ananda Mustafiz

Kazuo Nakashima

Samson Nalapalli

Somen Nandi

Marina Naoumkina

June B. Nasrallah

Kumari Neelam

vladimir nekrasov

Xinhui Nie

Yuese Ning

Long‐Jian Niu

Steve Nocker

Graham Noctor

Intawat Nookaew

Valentine Ntui

Tim O'Hare

Masanori Okamoto

Enrique Olmos

Donald Ort

Diego Orzaez

Anne Osbourn

Leon Otten

Katarzyna Otulak‐Kozieł

Paul Otyama

Therese Ouellet

Xinhao Ouyang

Yoshihiro Ozeki

Nam‐Chon Paek

Gabriel PAES

Ajay K Pandey

Manish Pandey

Sona Pandey

Chao‐You Pang

Xiaoming Pang

Roberto Papa

Youn‐Il Park

Isobel Parkin

Martin Parry

Raj Pasam

Katharina Paschinger

John Patrick

Matthew Paul

Katharina Pawlowski

Junhua Peng

Liangcai Peng

Xiaojian Peng

Xin‐Xiang Peng

Maria Penna

Joao Pennacchi

Lazaro Peres

Jose Manuel Perez‐Perez

Sambasivam Periyannan

Waranyoo Phoolcharoen

Lorcan Pigott‐Dix

Tomasz Pniewski

Marylou Polek

Orelvis Portal

Manoj Prasad

Pramod Prasad

Els Prinsen

Li Pu

Holger Puchta

Yiping Qi

Qian Qian

Kun Qiao

Feng Qin

Genji Qin

Jin‐Long Qiu

Li‐Juan Qiu

Yongfu Qiu

Feng Qu

Guan‐Zheng Qu

Qiudeng Que

Francisco R Tadeo

Arthur Ragauskas

Sadequr Rahman

Christine Raines

Mani Ramasamy

Xiaolan Rao

Keerti Rathore

James Rauschendorfer

Jochen Christoph Reif

Rajko Reljic

Claire Remacle

Wan‐Jun Ren

Matthew Reynolds

Dae‐Kyun Ro

Pedro Rodriguez

Adrienne Roeder

Emelda Rohani

Sergio Rosales‐Mendoza

Daniele Rosellini

Laura Rossini

Edward Rybicki

Avi Sadka

Cyrille Saintenac

Scott Sattler

Luc Saulnier

Rachit Saxena

Olga Sayanova

Michael Scanlon

Jodi Scheffler

Renate Scheibe

Henrik Vibe Scheller

Michael Schlappi

Karl Schmid

Thomas Schmuelling

Thorsten Schnurbusch

ARJEN SCHOTS

Christian Schulze Gronover

Robert Schuurink

Georg Seifert

Lorena Sendin

zeba seraj

Francesco Sestili

David Seung

Sergey Shabala

SHIHUA SHAN

Weixing Shan

Yi Shang

Thomas Sharkey

Anket Sharma

Robert Sharwood

Kumar Shashi

Anthony Shelton

Qian‐Hua Shen

Xinlian Shen

Huazhong Shi

Jiaqin Shi

Jinrui Shi

Tao Shi

Patrick Shih

Katsuhiro Shiratake

Jay Shockey

Tsubasa SHOJI

Fredy DA Silva

Stacy Singer

Manjit Singh

Rahul Singh

Sneh Singla‐Pareek

Nicholas Smirnoff

Alison Smith

Stuart Smyth

Amolkumar Solanke

Beng Kah Song

Chuankui Song

Chun‐Peng Song

Fengming Song

Guo‐qing Song

Shiyong Song

Xian‐Liang Song

Davide Sosso

Pierre Sourdille

Paul South

Erin Sparks

Nese Sreenivasulu

shivani srivastava

Dorothee Staiger

James Stangoulis

Florin Stanica

Neal Stewart

Eva Stoger

Helena Storchova

Steve Strauss

Simon Strobbe

Paul C. Struik

Robert Stupar

Jogaiah Sudisha

Shigeo S. Sugano

Sivakumar Sukumaran

Chuanqing Sun

Genlou Sun

Jie Sun

Shiyong Sun

Wenxian Sun

Francesco Sunseri

Nobuhiro Suzuki

Jeremy Sweet

W. Tadesse

Xiao‐Li Tan

Wakana Tanaka

Jihua Tang

Weihua Tang

Xiaoyan Tang

Li Tao

Xiaorong Tao

Miho Tatsuki

Ian Tetlow

Glenn Thorlby

Bala Thumma

Gregory Thyssen

Bin Tian

Feng Tian

Ji Tian

Zhixi Tian

Nicholas Tinker

Alain Tissier

Ratan Tiwari

Hongning Tong

Taiyo Toriba

Davoud Torkamaneh

Kay Trafford

Ben Trevaskis

Jaindra Tripathi

Horokazu Tsukaya

Lili Tu

Roberto Tuberosa

Gerald Tuskan

Alexey Tyunin

Yoshiaki Ueda

David Ullisch

BABAR USMAN

Dominique Van Der Straeten

Andre van Eerde

Steffen Vanneste

Serena Varotto

Felicity Vear

Veena Veena

Wilfred Vermerris

Silvere Vialet‐Chabrand

Yogesh Vikal

Nicolaus von Wiré

Jianmin Wan

Xiangyuan Wan

Cailin Wang

Daowen Wang

Ertao Wang

Guo‐Liang Wang

guodong wang

Hai Wang

Haiyang Wang

Hongxia Wang

Huanzhong Wang

Jia‐Wei Wang

Jiang Wang

Jianxin Wang

Kan Wang

Kejian Wang

Kun Wang

Linhai Wang

lirong Wang

Liyuan Wang

Nan Wang

Nian Wang

Wen Wang

Wen‐Ming Wang

Wenming Wang

Wenqin Wang

Wensheng Wang

X. Wang

Xianglan Wang

Xiaobao Wang

Xiaofeng Wang

Xiaojie Wang

Xingfen Wang

Xiyin Wang

Xuewen Wang

Yanpeng Wang

Yi Wang

Yong Wang

Yonghong Wang

Youping Wang

Yuanchao Wang

yucheng wang

Yuejin Wang

Zeng‐Yu Wang

Jianhua Wei

Peng‐Cheng Wei

Xin Wei

Bernd Weisshaar

Ralf Welsch

Tianwang Wen

Allan Wenck

James Whelan

Frank White

Thomas WIDIEZ

Iain Wilson

Margaret Woodhouse

David Wright

Changyin Wu

Jiahe Wu

Jianguo Wu

Jianyu Wu

jun wu

Keqiang Wu

Liuji Wu

Qiang Wu

Weiren Wu

Yongrui Wu

Enhua Xia

Guixian Xia

Lanqin Xia

Rui Xia

Xianchun Xia

Chengbin Xiang

Han Xiao

Shunyuan Xiao

Yingjie Xiao

Daoxin Xie

De‐Yu Xie

Kabin Xie

Wengang Xie

Xianrong Xie

Haiping Xin

Yongzhong Xing

Ai‐Sheng Xiong

Lizhong Xiong

Chenwu Xu

Fan Xu

Guohua Xu

Jianfeng Xu

Jin‐rong Xu

Juan Xu

Le Xu

Lingyang Xu

Mingliang Xu

Qiang Xu

Xiaodong Xu

Yangchun Xu

Reena Yadav

Richa Yadav

Kohji Yamada

changjie Yan

Jianbing Yan

Wenhao Yan

Bing Yang

Fusheng Yang

Jinghua Yang

Jun Yang

Jun Yang

Qing‐Yong Yang

Tae‐Jin Yang

Weibing Yang

Xiaohong Yang

Yongil Yang

Zhaoen Yang

Jia‐Long Yao

Wen Yao

Chuyu Ye

Heng Ye

Xudong Ye

Zheng‐Hua Ye

Keke Yi

Shuji Yokoi

Yordan Yordanov

Joshua Young

Deyue Yu

Diqiu Yu

Guohui Yu

Hong Yu

Jianming Yu

Jiwen Yu

Qingyi Yu

Meng Yuan

Zheng Yuan

Hu Yuxin

Lorenzo Zacarías

Keran Zhai

Jiang Zhang

Guangheng Zhang

Baohong Zhang

Chenming Mike Zhang

Chunbao Zhang

Chunyu Zhang

Dabing Zhang

Fei Zhang

Feng Zhang

Hong Zhang

Hui Zhang

Jianwei Zhang

Jie Zhang

Liangsheng Zhang

Lichao Zhang

Mingyong Zhang

Shuqun Zhang

Tao Zhang

Tianzhen Zhang

Xianlong Zhang

Xiaoming Zhang

Xingtan Zhang

Xingtan Zhang

Yang Zhang

Yanghanzi Zhang

Yingxiao Zhang

Yong Zhang

Yuanyuan Zhang

Yuelin Zhang

Zhanyuan Zhang

Zhengguang Zhang

Zhengsheng Zhang

Zhihong Zhang

Zhong‐Lin Zhang

Jian Zhao

Jianjun Zhao

Jiuran Zhao

Kaijun Zhao

Qiao Zhao

Qin Zhao

Qing Zhao

Yuanyuan Zhao

Yunde Zhao

Yunjun Zhao

Binglian Zheng

Tian‐Qing Zheng

Silin Zhong

Junhui Zhou

Man Zhou

Fei Zhou

Huanbin Zhou

Ji Zhou

Peng Zhou

Xiao Fan Zhou

Yihua Zhou

Yong Zhou

Zhengkui Zhou

Benzhong Zhu

Changfu Zhu

Guangtao Zhu

Hongliang Zhu

Jian‐Kang Zhu

Jian‐Kang Zhu

Qian‐Hao Zhu

Qiang Zhu

Qinlong Zhu

Yang Zhu

Ying Zhu

Chunliu Zhuo

Olga Zimina

Yuan Zong

Shimin Zuo

Jason Zurn

